# Epiphycan Predicts Poor Outcomes and Promotes Metastasis in Ovarian Cancer

**DOI:** 10.3389/fonc.2021.653782

**Published:** 2021-11-23

**Authors:** Lu Deng, Dandan Wang, Shouzhen Chen, Weiguo Hu, Ru Zhang

**Affiliations:** ^1^ Department of Gynaecology, The Hospital of Obstetrics & Gynaecology, Fudan University, Shanghai, China; ^2^ Shanghai Key Laboratory of Signaling and Disease Research, Laboratory of Receptor-Based Bio-Medicine, School of Life Sciences and Technology, Tongji University, Shanghai, China; ^3^ Department of Gynecology and Obstetrics, The Affiliated Suzhou Hospital of Nanjing Medical University, Suzhou Municipal Hospital, Suzhou, Jiangsu, China

**Keywords:** epiphycan, SLRPs, ovarian cancer, metastasis, invasion

## Abstract

The small leucine-rich proteoglycan (SLRP) family is widely expressed in extracellular matrix and aggravates tumor progression. However, epiphycan (EPYC), as a member of the SLRPs family, its biological function in cancer has not been confirmed. Thus, we aimed to clarify the role of EPYC in progression of ovarian cancer (OC), and further analyze the molecular mechanisms implicated in tumorigenesis. Here, we analyzed the differential expression genes of GSE38734, including 4 matched primary OC and metastatic tissues. We obtained OC RNAseqs data from the Cancer Genome Atlas (TCGA) and analyzed the correlation between EPYC expression and OC staging, pathological grading, etc. The expression of EPYC in OC and normal ovarian tissues was compared in Oncomine website. We used siRNAs to interfere the expression of EPYC in ovarian cancer cell line SKOV3. Scratch test, transwell-matrigel chamber, CCK8 assay were used to detect the changes of SKOV3 migration, invasion and proliferation ability after EPYC was interfered. We used R software to make GO and KEGG analysis of related genes of EPYC. We used the Hitpredict website to predict interacting proteins. The results showed that the expression of EPYC in metastatic ovarian cancer was higher than primary ovarian cancer, and that in primary cancer was higher than normal ovaries. After siRNA interferes with EPYC expression, the migration, invasion and proliferation of SKOV3 cells were weakened. EPYC mainly played a role in ECM organization, and involved in PI3K/Akt, focal adhesion signaling pathways. EPYC might interact with PLCG2 and CRK, and be involved in signal transduction.

## Introduction

Ovarian cancer (OC) is one of the three major malignant tumors of female reproductive system, more than 90% of which is epithelial ovarian cancer, and its mortality rate ranks first among the three major gynecological malignancies (1). Although surgical techniques have been continuously improved in recent years and new drugs have been emerging, the 5-year survival rate of ovarian cancer patients has been hovering at 30%-40% in the past 30 years (2). The main reason is that the pathogenesis of ovarian cancer is hidden and lacks specific symptoms at early stage. 70% of the patients are at an advanced stage when they see a doctor, accompanied by extensive metastasis and abdominal dissemination (3). The extensive metastasis and spread of ovarian cancer are related to the invasion and metastasis ability of cancer cells. However, the mechanism of ovarian cancer cell invasion and metastasis is still unclear. Therefore, there is an urgent need to go further research to explain the molecular changes in the process of invasion and metastasis of ovarian cancer, to explore new biomarkers and potential targets related to ovarian cancer invasion and metastasis, so as to improve the diagnosis, treatment and prognosis of patients with ovarian cancer.

Small leucine-rich repeat proteoglycan (SLRP) family is widely present in the extracellular matrix (ECM), participating in matrix formation, and potentially regulate cancer cell proliferation, angiogenesis and migration (4). There are 18 members in SLPRs family. Based on their protein structures, they are divided into 5 subtypes. The most studied types are I-III subtypes. Decorin (DCN) is one of type I SLPRs. It plays a protective role in tumors mostly. In breast cancer cells, DCN restricts cancer cell proliferation and induces cell differentiation by down-regulating the receptor tyrosine kinase human epidermal growth factor receptor 2 (HER-2) (5). DCN inhibits tumor growth through anti-vascular effects (6). By inactivating c-met, the downstream beta-catenin signaling pathway is inhibited, thereby DCN inhibits the spread of cancer (7). Biglycan (BGN) is another type I SLRPs, which is widely present in the ECM and serves as the main matrix component and key signal molecule. The expression of BGN in tumor stroma is up-regulated and is related to cell proliferation, cell migration, metastasis and angiogenesis (8). BGN interacts with TLR2 and TLR4 and promotes gastric cancer cell migration through the TLR/NF-kappaB/HIF1-alpha regulatory axis (9). The upregulation of BGN in colon cancer cells induces NF-κB to mediate cell chemoresistance, and to reduce cell apoptosis (10). Lumican (LUM) is one of the type II SLRPs, and its expression is significantly increased in a variety of tumors, especially lung cancer, gastric cancer, colon cancer, pancreatic cancer and bladder cancer (11–15). LUM is highly expressed in fibroblasts in gastric cancer Cells (CAF) and regulates the FAK signaling pathway by activating integrin beta 1 to promote cancer cell dissemination (11). Epiphycan (EPYC), also known as Dermatan sulfate proteoglycan 3 (DSPG3), proteoglycan-Lb (PG-Lb), is a proteoglycan and one of type III SLRPs. Its gene contains 7 exons, and exons 3 and 7 contain potential glycosaminoglycan attachment sites (16), which can interact with collagen fibers and other ECM proteins to promote the formation of type I collagen fibers (17). EPYC is expressed in cartilage, ligament, placenta and other tissues (18), and plays an important role in cartilage development and joint integrity maintenance (19). Lack of expression of EPYC can promote hearing impairment (20) and corneal dystrophy (21). However, there are very few studies on the role of EPYC in tumors. Until 2019, Mariani et al. (22)performed the transcript sequencing of ovarian cancer and intestinal metastasis of ovarian cancer, and found that EPYC and LUM were two of the up-regulated genes in intestinal metastasis ovarian cancer tissues and related to prognosis. In patients who achieved complete tumor reduction surgery, only EPYC expression level was still associated with poor prognosis. It indicates that EPYC may play an important role in the malignant progression of ovarian cancer. However, the function and mechanism of EPYC in ovarian cancer are still unknown.

Thus, in the present study, we compared the expression of EPYC in metastatic OC and primary OC by analyzing expression profiles of GSE38734 (23). Then, we explored the effects of EPYC on the proliferation, invasion and metastasis of OC by interfering with EPYC expression. At last, the possible mechanism of EPYC was preliminarily discussed.

## Materials and Methods

### Public Databases Analysis

The differential genes expressed in ovarian cancer and metastatic ovarian cancer were downloaded from GEO database (https://www.ncbi.nlm.nih.gov/geo/) under the accession number GSE38734. Differential expression genes of GSE38734 were analyzed by R software using *limma* package. We downloaded the raw data of TCGA database from UCSC Xena (https://xena.ucsc.edu/). LinkedOmics is a publicly available platform for analyzing multiomics data from TCGA database (http://www.linkedomics.org/login.php). We searched for interacted proteins of EPYC on website of HitPredict (http://www.hitpredict.org/). Genes that correlated with EPYC in OCs were obtained from this website, then the corresponding genes were subjected to Gene Ontology (GO) functional analysis and Kyoto Encyclopedia of Genes and Genomes (KEGG) pathway by R software using *clusterProfiler* package, as well as the GSEA analysis. Genes correlated with EPYC was analyzed by R software using Pearson correlation analysis.

### Cell Culture and siRNA Transfection

All ovarian cancer cell lines and normal human ovary cells were purchased from Fuheng Biology and cultured in a humidified atmosphere of 5% CO2 at 37°C. Cell lines were cultured in RPMI 1640 medium. A total of 10% fetal bovine serum (FBS) was supplemented in the culture medium. EPYC-siRNAs, as well as the corresponding negative control, were synthesized from RibBio (Guangzhou, China). siRNA transfection were performed using the matching siRNA transfection kit from RibBio (Guangzhou, China).

### RNA Extraction, Reversely Transcribed PCR, and Quantitative Real-Time Polymerase Chain Reaction

TRIzol (Invitrogen, Carlsbad, CA, USA) was used to extract the total RNA from cells. According to the protocol, total RNA was reversely transcribed into cDNA using the two-step reverse transcription reagents (Promega, UK). SYBR Green Master (ROX) (Bimake.com) was used for qRT-PCR according to the protocol. The relative gene expression of EPYC, *SNAI2*, *CDH1* was calculated by the method of 2−ΔΔCt with *ACTB* gene expression as a control. The primer sequences of *EPYC*, *SNAI2*, *CDH1* and *ACTB* were shown below (**Table 1**).

**Table 1:** The primer sequences.

Primers	Sequences
EPYC-forward	ATTAGCAGGACTTGTTCT
EPYC-reverse	TTCTAAGGTGGCATCATA
SNAI2-forward	CTGTGACAAGGAATATGTGAGC
SNAI2-reverse	CTAATGTGTCCTTGAAGCAACC
CDH1-forward	AGTCACTGACACCAACGATAAT
CDH1-reverse	ATCGTTGTTCACTGGATTTGTG
ACTB-forward	GGCCAACCGCGAGAAGATGAC
ACTB-reverse	GGATAGCACAGCCTGGATAGCAAC

### Western Blot

RIPA lysis buffer (beyotime, catlog# P0013B) was used to extract total protein of cells. BCA assay kit (Thermo Fisher Scientific, catlog#23225) was used for protein quantification. Then, equal amount of proteins were loaded for SDS-PAGE electrophoresis. After transferring to PVDF membranes, the membranes were blocked with 5% skim milk for 1.5 hours. After milk blocking, the membrans were incubated with EPYC antibody or β-actin antibody at 4°C overnight (EPYC antibody: neuromics, catlog# MO15127, β-actin antibody: proteintech, catlog# 60008-1-Ig). After incubating with horseradish peroxidase (HRP)-conjugated secondary antibody (CWBIO, catlog# CW0102) for 2 hours, protein bands were visualized using the enhanced chemiluminescence detection kit (Thermo Fisher Scientific, catlog# 34095) under the Chemiluminescence system.

### Wound Healing Assay

1*10^5 cells/well were seeded into 24-well plates and incubated for 12 hours to make cells adhere to the wall. Then EPYC-siRNAs and negative control were added into culture medium according to the transfection protocol. After interfering for 24 hours, we used a 200 ul pipette tip to make a “cross-shaped” wounds. After gently washing 3 times with PBS, cells were cultured in RIPM 1640 medium containing 2% FBS for another 24 hours and images were captured under a phase-control microscope.

### Transwell Assay

SKOV3 cells were seeded and transfected as described above. 24 hours later, the transfected cells were resuspended in serum-free RIPM1640 medium and added to the upper chambers of the transwell chamber (Corning, NY, USA). Add RIPM1640 containing 10% FBS to the lower chamber to induce cell invasion. After culturing for 24 hours, the bottom of upper chambers were fixed by 4% paraformaldehyde for 30 minutes and then stained with 0.5% crystal violet for 30 minutes, and the cells on the upper surface of the membrane were removed by a cotton swab. After washing and the chamber is air-dried, the membranes of the bottom of upper chamber were captured under a microscope for analysis.

### CCK8 Assay

Cell counting kit-8 (CCK8) (Beyotime, Shanghai, China) was applied in detecting proliferation of SKOV3 cells after transfecting EPYC-siRNAs and negative control for 0, 24, 48, 72 hours. We operated according to the instructions.

### Statistics

Statistical analysis was performed using R software. 2-3 experiments were repeated in our study. Data was presented as mean ± standard deviation (SD), and two-tailed *t*-test was utilized to compare the difference between two independent samples. The log-rank test was used for Kaplan–Meier survival analysis. *P* < 0.05 was considered statistically significant.

## Results

### EPYC Is Upregulated in Metastatic Lesions of OCs

**Figure 1:**
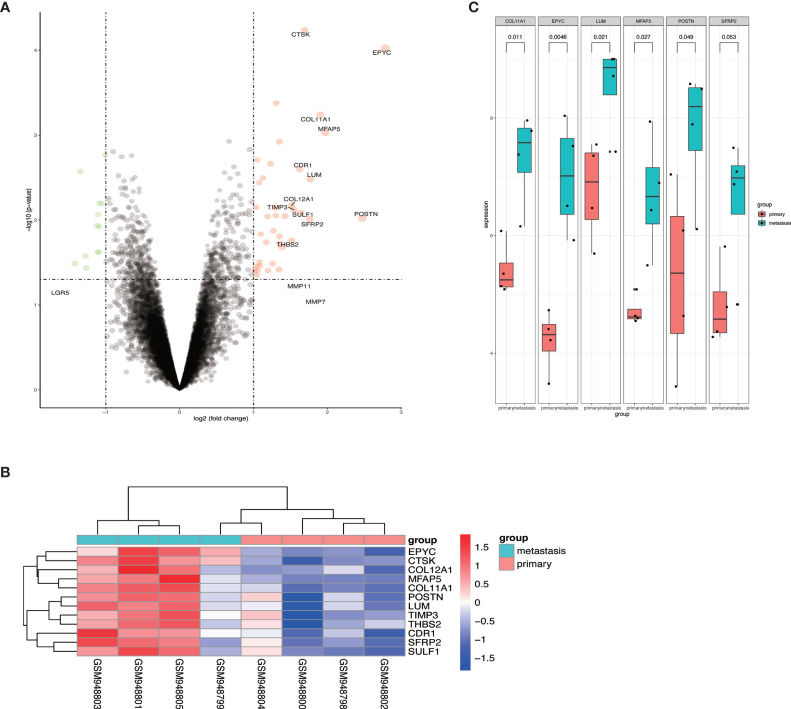
The expression of EPYC in metastatic ovarian cancer tissue was significantly increased. **(A, B)** Volcano map and heat map of differential genes from GSE38734 dataset; **(C)** The top 6 up-regulated genes in ovarian metastatic cancer compared with paired OC.

To identify the differential expressed genes in metastatic OCs, we analyzed the expression profiles of GSE38734. The datasets contained the mRNA sequencing of 4 primary OCs and their metastatic OC tissues. Differentially expressed genes (DEGs) were analyzed by using R software, and found out that EPYC was the most elevated gene in metastatic OC (**Figures 1A–C**).

### EPYC Is Obviously Up-Regulated in Ovarian Cancer

**Figure 2:**
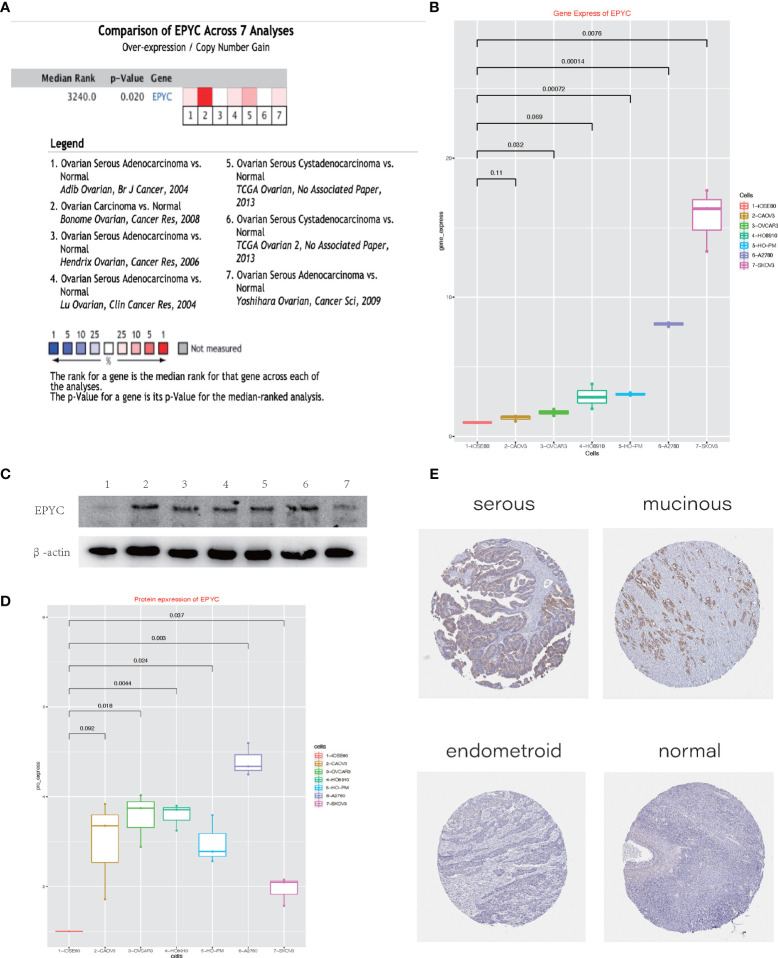
The expression of EPYC in ovarian cancer is significantly increased. **(A)** Oncomine website meta-analysis of EPYC expression in normal ovarian tissues and ovarian cancer tissues. **(B)** Real-time PCR to detect the expression of EPYC in ovarian cancer cells and normal ovarian cells. **(C, D)** Western-blot detects the expression of EPYC in ovarian cancer cells and normal ovarian cells. **(E)** Expression of EPYC protein in normal ovary and different subtypes of OCs (Image source: The HPA website, antibody: HPA045455).

To explore the role of EPYC on progression of OCs, we compared the expression of EPYC in OCs and normal ovary. Totally, 7 studies comparing the expression of EPYC in OCs and normal ovary tissues were identified in an online website ONCOMINE. EPYC expression was significantly up-regulated in ovarian cancers (p=0.02) (**Figure 2A**). Furthermore, we compared the gene and protein expression of EPYC in normal ovarian cells and several ovarian cancer cells. We found that the endogenous EPYC expression was higher in OC cells than normal ovary cells, IOSE80. The expression of EPYC mRNA was significantly higher in A2780 and SKOV3 than the other OC cells (**Figure 2B**). By using western blot, we detected the protein expression level of EPYC in normal ovarian cell and OC cells. The result showed that EPYC protein level was higher in OC cells than in normal ovarian cells (**Figures 2C, D**).

The human protein atlas (HPA) is a Swedish-based program initiated in 2003 for mapping all the human proteins in cells, tissues and organs (https://www.proteinatlas.org/). From the HPA website, we learned that EPYC protein was barely expressed in normal ovarian tissue (0/3), but could be moderately expressed in a third of OC tissues (4/12, 2 serous cystadenocarcinomas and 2 mucinous cystadenocarcinomas). All three endometroid OCs were negative (**Figure 2E**).

### EPYC Accelerates Invasion and Metastasis of OC Cells *In Vitro*

**Figure 3:**
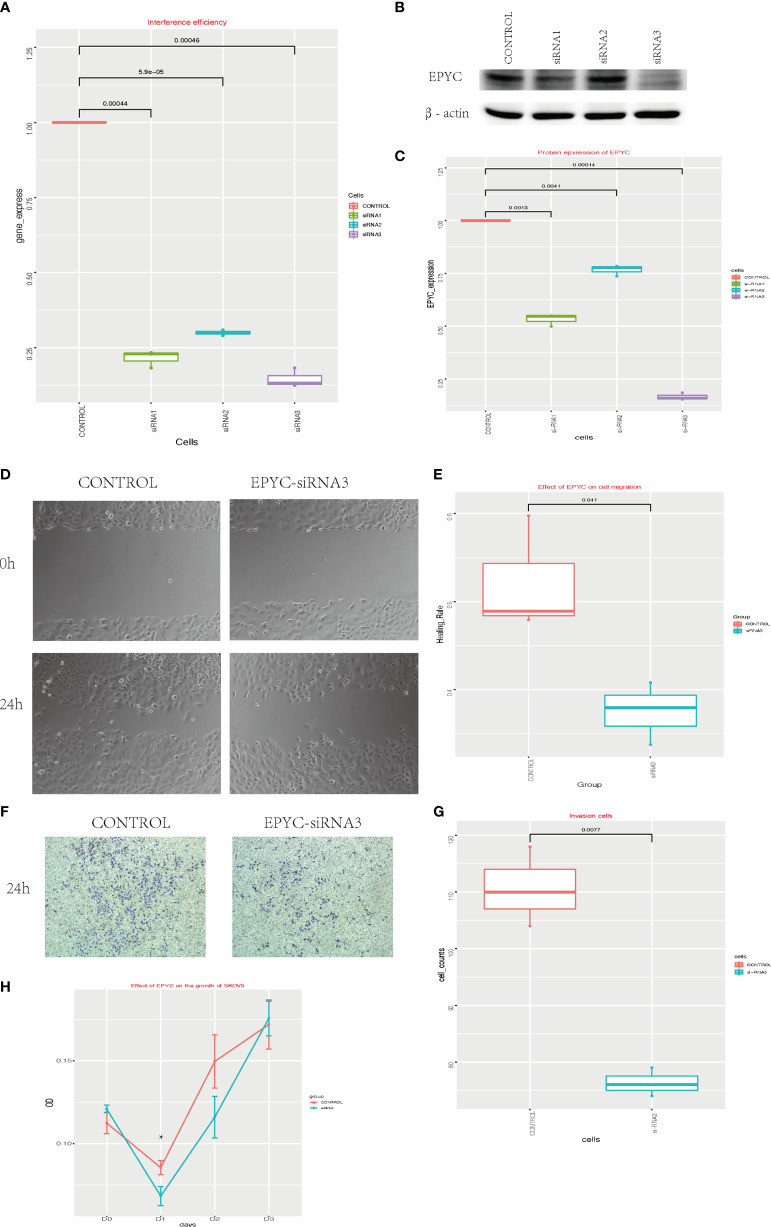
The function of EPYC. **(A)** EPYC mRNA expression detected by real-time qPCR after EPYC-siRNAs interfering. **(B, C)** EPYC protein expression detected by western blot. **(D, E)** Effect of EPYC on SKOV3 migration. **(F, G)** Effect of EPYC on SKOV3 invasion. **(H)** Effect of EPYC on SKOV3 growth. * means after 24 hours, the OD value was significantly lower in EPYC-siRNA group than the control group, detected by CCK8, p < 0.05.

To explore the function of EPYC in ovarian cancer, we interfered the expression of EPYC in SKOV3 cells by EPYC-siRNAs. We verified the interference efficiency by qRT-PCR experiment and western blot (**Figures 3A–C**). Subsequently, we explored the effect of EPYC on the motility of OC cells. In the scratch test, we found out that after EPYC interfering, the migration ability of SKOV3 was obviously impaired (**Figures 3D, E**). In the transwell-matrigel assays, EPYC-siRNA significantly impaired the invasion ability of SKOV3 cells (**Figures 3F, G**).

### EPYC-siRNA Inhibits Cell Growth of OC *In Vitro*

We also explored the function of EPYC on cell growth of OC cells by CCK-8 assays. EPYC-siRNA suppressed the growth of SKOV3 cells after interfering for 24 hours, but then the effect diminished (**Figure 3H**).

### Enrichment Analysis of EPYC Functional Networks in Ovarian Cancer

**Figure 4:**
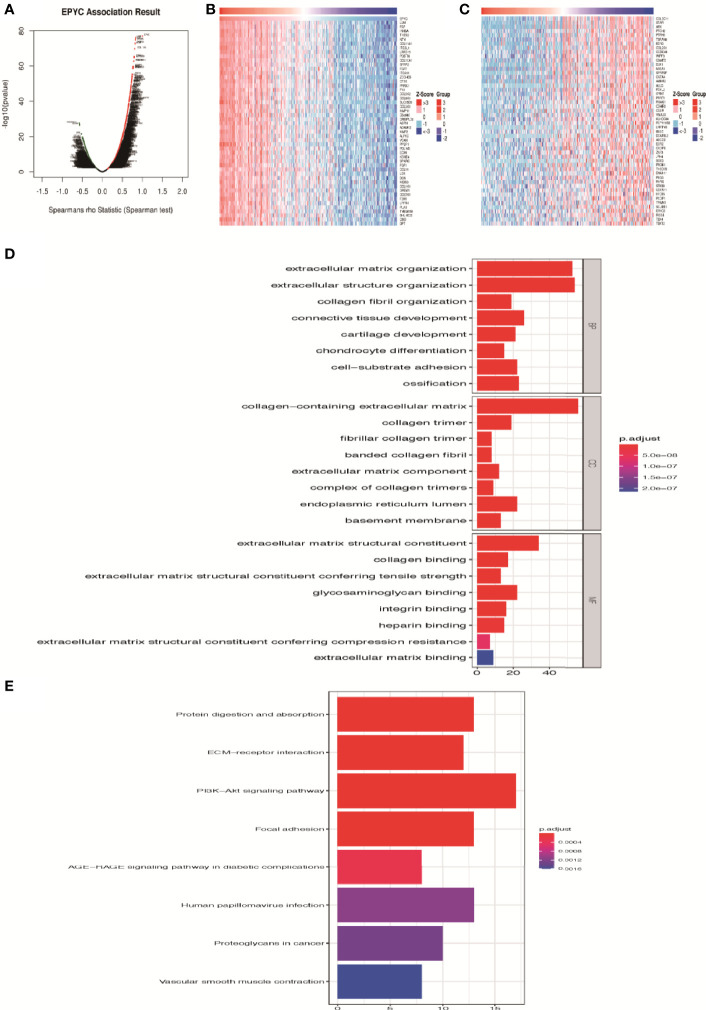
Enrichment analysis of EPYC functional networks in ovarian cancer. **(A)** EPYC co-expressing genes in OCs were analyzed by spearson test (LinkedOmics). **(B, C)** Top 50 genes positively/negatively correlated with EPYC expression in OCs (LinkedOmics). **(D)** Top 8 elements significantly enriched in GO categories of top 200 genes positively correlated with EPYC expression. **(E)** KEGG pathway analysis of top 200 genes positively correlated with EPYC expression.

On the website LinkedOmics (24), we analyzed the most related genes of EPYC using 581 ovarian cancer patients data from TCGA database. As shown in the association curve (**Figure 4A**), 4,443 genes (red dots) showed significantly positive correlation with EPYC, whereas 5,121 genes (green dots) showed significantly negative correlation with EPYC(p-value<0.05). The top 50 genes of both were shown in the heatmap (**Figures 4B, C**). Significant GO term analysis using ClusterProfiler package (25) by R software showed that top 200 genes significantly positively correlated with EPYC primarily participated in extracellular matrix (ECM) organization, collagen binding, and ECM constituent conferring tensile strength. They could perform the molecular function of ECM structural constituents, collagen binding, glycosaminoglycan binding and integrin binding (**Figure 4D**). In the KEGG pathway analysis of these genes, we found that they were significantly enriched in the following pathways: PI3K-Akt signaling pathway, focal adhesion, protein digestion and absorption and ECM-receptor interaction (**Figure 4E**).

### EPYC May Interact With PLCG2 and CRK

**Figure 5:**
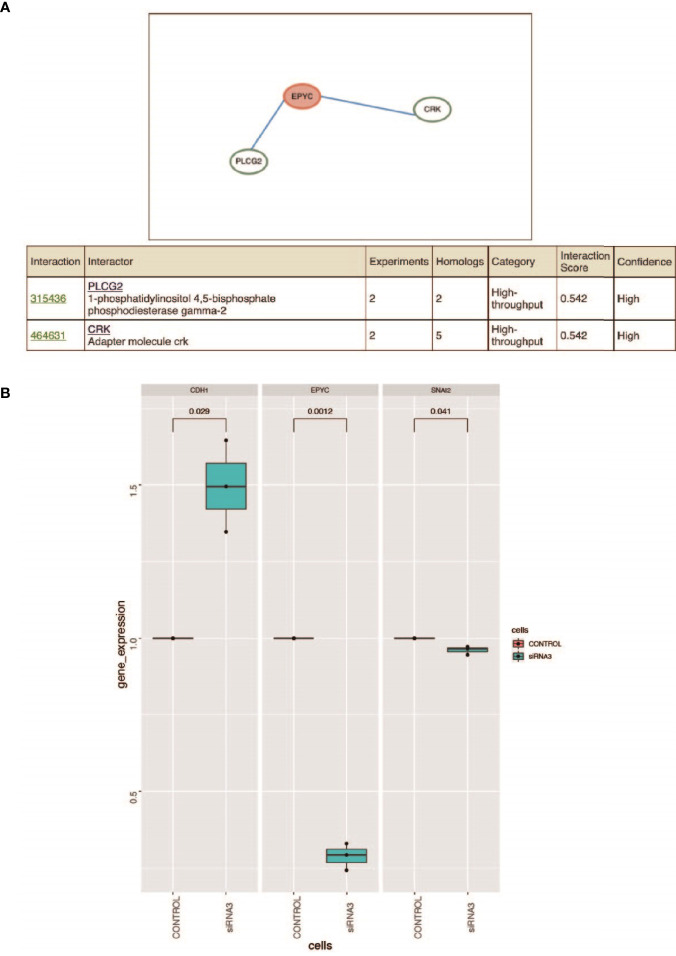
**(A)** Prediction of the interacted proteins of EPYC on HitPredict website. **(B)** After siRNA interferes with the expression of EPYC, the expression of SNAI2 is down-regulated and the expression of CDH1 is up-regulated. Changes in the expression of SNAI2 and CDH1 after the down-regulation of EPYC expression. Using qRT-PCR, it was found that after siRNA interfered with EPYC expression, SNAI2 expression was down-regulated, while CDH1 expression was significantly up-regulated.

HitPredict website is a database of quality assessed protein-protein interactions (26). We searched for the proteins interacting with EPYC. The results showed that phospholipase Cg2 (PLCG2) and CRK proto-oncogene, also named adaptor protein (CRK, or p38, or CRKII), were two proteins interacting with EPYC with high interaction score (**Figure 5A**). PLCG2 is a transmembrane signaling enzyme which catalyzes the conversion of PIP2 into second messengers IP3 and DAG by utilizing calcium catalysis. They initiate intracellular calcium flux and activate protein kinase C, respectively. PLCG2 is highly expressed in cells of hematopoietic origin and is responsible for regulating immune responses, platelet adhesion and spreading (26). CRK is a member of adapter protein families, and it has been implicated in many signal transduction events due to its SH2 and SH3 domains (27). SH2 interacts with phosphotyrosine, which results in CRK recruiting cytoplasmic proteins around tyrosine kinase (28). CRK has been shown to play a role in cancer progression and malignant behavior (29). CrkII is an alternatively spliced isoform of CRK, and could interact and be phosphorylated by insulin-like growth factor (IGF) receptor. The phospholated Crk may be related with the interference of IGF-1 regulatory pathway (30). The predicted interaction to these 2 proteins means EPYC maybe play important roles in regulating signal transduction and further experimental verification is needed.

### EPYC Might Mediate SNAI2 to Suppress the Expression of CDH1

It is a key step in the occurrence of EMT that SNAI2 as a transcription inhibitor directly down-regulates the expression of CDH1, and the TGFβ signaling pathway can regulate the expression of SNAI2 through SMAD pathway and the non-SMAD pathway (31). The PI3K/AKT pathway is one of the non-SMAD signaling pathways for TGF-β to induce epithelial-mesenchymal transition (EMT), which can regulate the metastasis of cancer cells (32).

Using the TCGA public database, we performed Gene Set Enrichment Analysis (GSEA) analysis. Gene enrichment analysis showed that EPYC might activate EMT, TGF-β and PI3K/AKT signals pathways (**Supplementary Figure 1**). Correlation analysis revealed that EPYC was significantly correlated with the expression of multiple TGF-β signaling pathway molecules (TGFB1, TGFBI, TGFB3, TGFBR1, TGFBR2, TGFBR3) (**Supplementary Figure 2**). After siRNA-EPYC interference, the invasion and metastasis ability of ovarian cancer cells was weakened, while the expression of SNAI2 was down-regulated and the expression of CDH1 is increased (**Figure 5B**).

Therefore, we speculated that EPYC in ovarian cancer might regulate the expression of SNAI2 by activating the TGF-β/PI3K/AKT signaling pathway, and promoted the occurrence of EMT and invasion and metastasis of ovarian cancer.

### EPYC High Expression Correlated With Poor Prognosis of OC Patients

**Figure 6:**
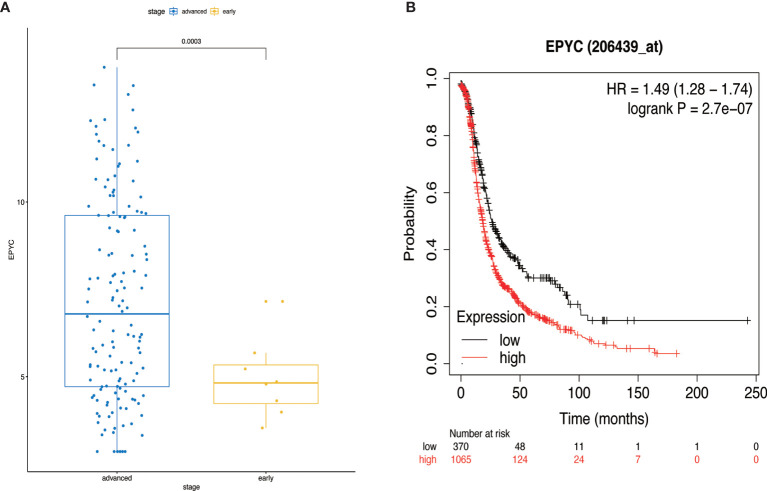
The clinical significance of EPYC. **(A)** When the EPYC expression level of advanced ovarian cancer was compared with that of early ovarian cancer, the expression level of advanced ovarian cancer was also significantly higher than that of early ovarian cancer. **(B)** The high expression of EPYC in serous ovarian cancer was associated with poor prognosis, analyzing by Kaplan-Meier Plotter website.

Using the TCGA database, we found out that EPYC gene expression was associated with late FIGO staging of ovarian cancer, without correlation with age, pathological grade, or death (**Figure 6A** and **Table 2**). The expression of EPYC and overall survival time of OC is not correlated using TCGA database, maybe because limited patients were included. However, when using the online tool Kaplan-Meier Plotter website, which includes 1435 OC patients, we found that EPYC expression was associated with poor prognosis of serous ovarian cancer, with cutoff value 23 used in analysis (**Figure 6B**).

**Table 2:** Expression of EPYC with clinicopathological parameter of ovarian cancers from the TCGA database.

	EPYC High (n = 283)	EPYC Low (n = 284)	p-value
Age			
Age<50	64 (22.6%)	50 (17.6%)	0.167
Age≥50	219 (77.4%)	234 (82.4%)	
FIGO stage			
Advanced	269 (95.1%)	251 (88.4%)	0.00635
Early	14 (4.9%)	33 (11.6%)	
Pathologic grade			
G1-G2	44 (15.5%)	31 (10.9%)	0.133
G3	239 (84.5%)	253 (89.1%)	
Event			
Yes	175 (61.8%)	164 (57.7%)	0.391
No	108 (38.2%)	119 (41.9%)	
Missing	0 (0%)	1 (0.4%)	

## Discussion

Tumor microenvironment, mainly including cellular components (including endothelial cells, immune cells, fibroblasts, pericytes, etc.), ECM components (including fibrous proteins, proteoglycans, minerals and secretion molecules) and some exosomes (33), establish an autocrine-paracrine communication circuit, through mutual signaling enhances the invasion and transfer of cancer cells (34). ECM, used to be considered as a support structure for tissues, is actually a dynamic structure, and its biomechanical characteristics regulate important processes of cancer development, such as migration, adhesion, proliferation and differentiation (33). Different tissues have different ECM compositions and display specific features (35).

SLRPs, a component of ECM, exist in a wide range of connecting tissues, and share common multiple Leucine-Rich Repeats (LRRs) (36). SLRPs are key matrix proteoglycan and have vital role in regulating cancer progression (4). DCN, BGN, LUM and fibromodulin (FMOD) are the most reported members of SLRPs involved in cancer progression (4, 37). They play a role in promoting or suppressing cancer progression in tumors through different pathways. The roles of DCN, BGN and LUM have been described above. FMOD plays vital roles in angiogenesis, TGF-β activity regulation, fibroblasts differentiation, apoptosis and metastasis (37). One of the roles of SLRPs is to interact with collagen fibrils to form protective coat to protect it from MMPs (MMP1 and MMP13)cleavage (38). Besides collagen, SLRPs also interact with various cytokines, including transforming growth factor-β1 (TGF-β1), bone morphogenic proteins, von Willebrand factor, platelet-derived growth factor (PDGF) and tumor necrosis factor-α (36). The interaction between TGF-β and DCN can occur while DCN binds to collagen, which prevents TGF-β binding to its receptors (39). By downregulating the bioactivity of TGF-β1, decorin counteracted the transcriptional repression of PDCD4 *via* inhibition of miRNA-21. Then, PDCD4 suppressed anti-inflammatory mediators such as IL-10, which creates a proinflammatory tumor microenvironment, thus retarding tumor growth (40, 41). In contrast to DCN, BGN is considered as a pro-angiogenic SLRP, as it binds to VEGFA and subsequently activates the VEGFR2 signaling pathway (42).

In the present study, we found that EPYC was the most overexpressed gene in metastatic ovarian cancer tissues than ovarian cancer by analyzing the GSE38734 raw data. Meanwhile, the expression of EPYC in OC was higher than that in normal ovaries. These results indicated that EPYC may play an important role in the development of OC, which was further confirmed in a subsequent functional experiment in OC cells. Similar to other SLRP members, EPYC has a high correlation with the expression levels of MMPs, CXCLs, and TGF-β (**Supplementary Table 1**). GO analysis showed that EPYC mainly plays a role in the arrangement of extracellular matrix. KEGG analysis indicated that EYPC may play a role through PI3K/AKT, FOCAL adhesion and other signaling pathways. PI3K/AKT pathway is frequently activated in human cancers and has been considered as a promising therapeutic target. The axis contributes to oncogenic transformation include stimulation of cell proliferation and survival, metabolic reprogramming, suppressing autophagy and senescence, invasion/metastasis and EMT (43). What’s more, EPYC has high interaction scores with PLCG2 and CRK, both of which are key signal transduction molecules.

EPYC is a relatively new member of SLRPs. EPYC may affect the biological behavior of OC through a variety of ways, and play an important role in the progression of OC. It is a potential candidate for diagnosis and treatment of ovarian cancer. In future studies, we will further explore and verify the mechanism involved in the action of EPYC.

## Data Availability Statement

The datasets presented in this study can be found in online repositories. The names of the repository/repositories and accession number(s) can be found in the article/**Supplementary Material**.

## Author Contributions

LD wrote the manuscript, conducted experiments, and analyzed data. DW analyzed results, and designed the manuscript structure. SC was responsible for public data analysis. WH and RZ modified the manuscript. All authors contributed to the article and approved the submitted version.

## Funding

This study was funded by National Natural Science Foundation of China (grant number: 81902642) and Basic Research on Hygiene Application in Suzhou City (grant number: SYS2019099).

## Conflict of Interest

The authors declare that the research was conducted in the absence of any commercial or financial relationships that could be construed as a potential conflict of interest.

## Publisher’s Note

All claims expressed in this article are solely those of the authors and do not necessarily represent those of their affiliated organizations, or those of the publisher, the editors and the reviewers. Any product that may be evaluated in this article, or claim that may be made by its manufacturer, is not guaranteed or endorsed by the publisher.
