# Supplementary material for: Epiphycan Predicts Poor Outcomes and Promotes Metastasis in Ovarian Cancer
Source: Front Oncol. 2021 Nov 23;11:653782. doi: 10.3389/fonc.2021.653782 (PMC8650094; doi:10.3389/fonc.2021.653782)

Supplemetary Figure 1.Gene lists were sorted by correlation and ranked Gene Set Enrichment Analysis (GSEA) was performed. A. GSEA enrichment analysis shows that EPYC-related genes positively activate the EMT pathway; B. GSEA enrichment analysis shows that EPYC-related genes are significantly enriched in the PI3K/AKT signaling pathway and positively activate the pathway; C. GSEA enrichment Set analysis showed that EPYC-related genes were significantly enriched in the TGF-β signaling pathway and positively activated the pathway.

A

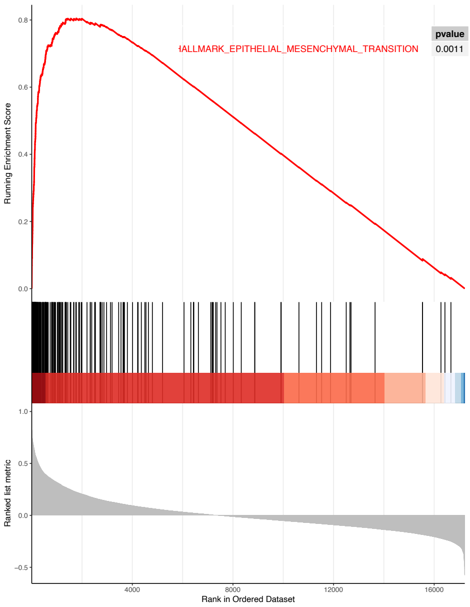

B

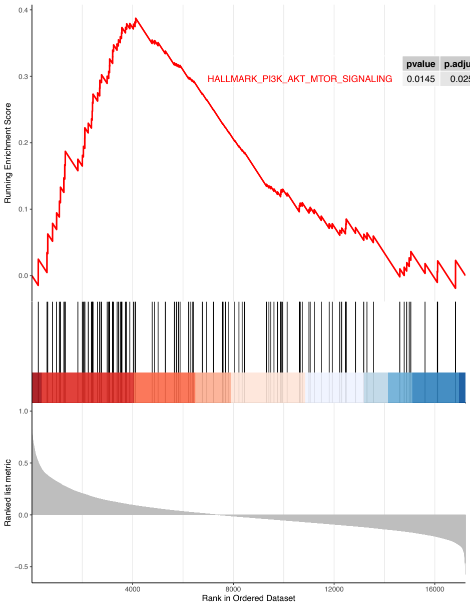

C

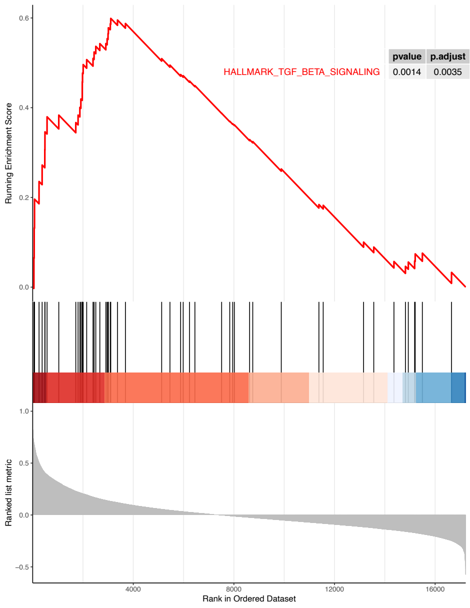

Supplemetary Figure 2. EPYC has a strong correlation with TGFβ signaling pathway molecules and SNAI2 expression. A. The Pearson correlation coefficient between EPYC and TGFB1 is 0.389, p<0.001; B. The Pearson correlation coefficient between EPYC and TGFBI is 0.506, p<0.001; C. The Pearson correlation coefficient between EPYC and TGFB3 is 0.445, p<0.001 ; D. Pearson correlation coefficient between EPYC and TGFBR1 is 0.332, p<0.001; E. Pearson correlation coefficient between EPYC and TGFBR2 is 0.391, p<0.001; F. Pearson correlation coefficient between EPYC and TGFBR3 is -0.214, p < 0.001; G. The Pearson correlation coefficient between EPYC and SNAI2 is 0.607, p < 0.001.

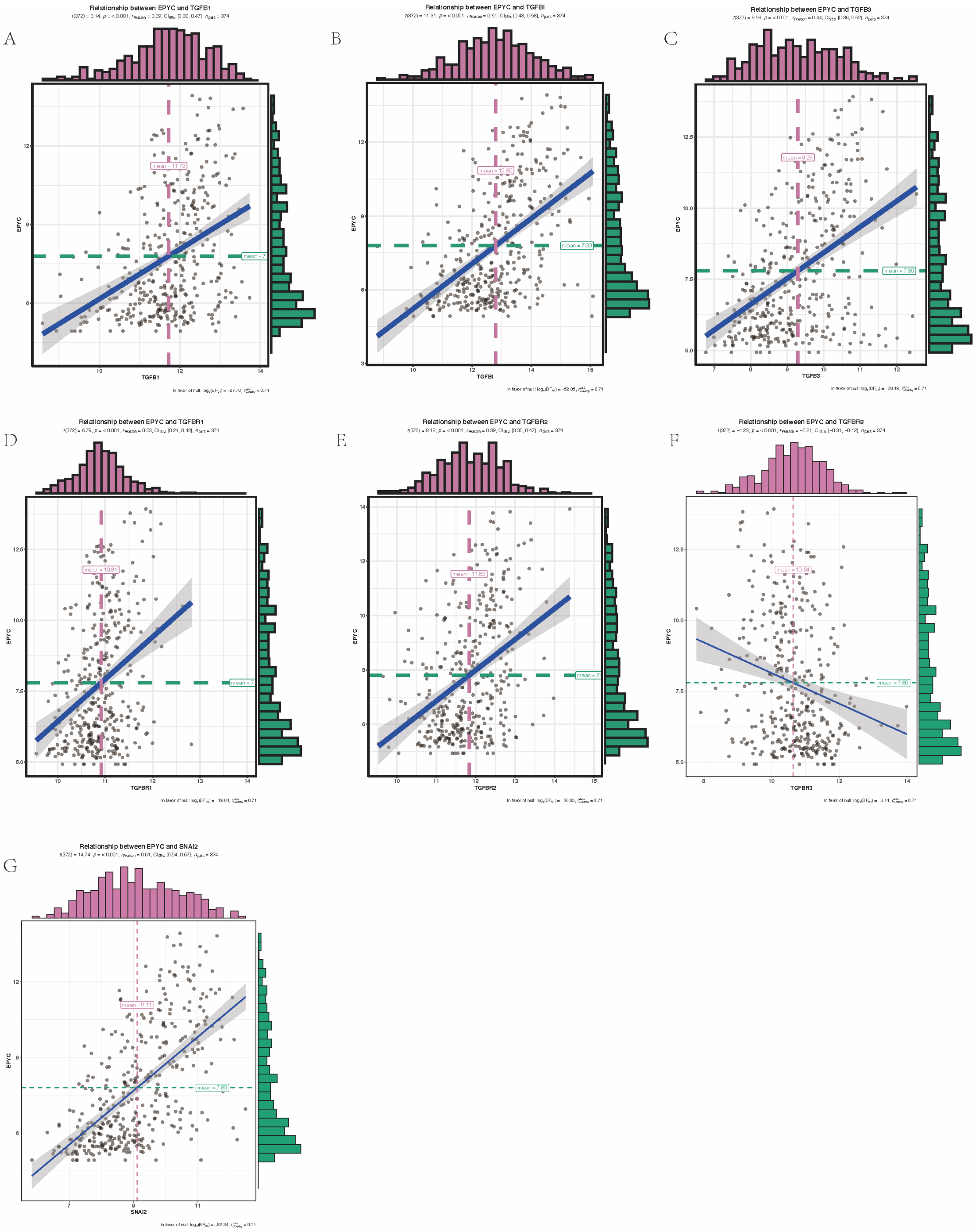

Supplement: Supplementary file 1 [file DataSheet_1.pdf]
